# Adrenomedullin 2 improves bone regeneration in type 1 diabetic rats by restoring imbalanced macrophage polarization and impaired osteogenesis

**DOI:** 10.1186/s13287-021-02368-9

**Published:** 2021-05-13

**Authors:** Feng Wang, Lingchi Kong, Wenbo Wang, Li Shi, Mengwei Wang, Yimin Chai, Jia Xu, Qinglin Kang

**Affiliations:** Department of Orthopaedic Surgery, Shanghai Jiao Tong University Affiliated Sixth People’s Hospital, Shanghai, 200233 People’s Republic of China

**Keywords:** Adrenomedullin 2, Diabetes mellitus, Bone regeneration, Macrophage polarization, Bone marrow mesenchymal stem cell, Distraction osteogenesis

## Abstract

**Background:**

Both advanced glycation end products (AGEs) and AGE-mediated M1 macrophage polarization contribute to bone marrow mesenchymal stem cell (BMSC) dysfunction, leading to impaired bone regeneration in type 1 diabetes mellitus (T1DM). Adrenomedullin 2 (ADM2), an endogenous bioactive peptide belonging to the calcitonin gene-related peptide family, exhibits various biological activities associated with the inhibition of inflammation and reduction of insulin resistance. However, the effects and underlying mechanisms of ADM2 in AGE-induced macrophage M1 polarization, BMSC dysfunction, and impaired bone regeneration remain poorly understood.

**Methods:**

The polarization of bone marrow-derived macrophages was verified using flow cytometry analysis. Alkaline phosphatase (ALP) staining, ALP activity detection, and alizarin red staining were performed to assess the osteogenesis of BMSCs. Quantitative real-time polymerase chain reaction, enzyme-linked immunosorbent assay, western blotting, and immunofluorescence staining were used to assess polarization markers, nuclear factor kappa-light-chain-enhancer of activated B cells (NF-κB) signaling, and osteogenic markers. In vivo, a distraction osteogenesis (DO) rat model with T1DM was established, and tibia samples were collected at different time points for radiological, biomechanical, and histological analyses, to verify the effects of ADM2 on bone regeneration and M2 polarization under diabetic conditions.

**Results:**

ADM2 treatment reversed AGE-induced M1 macrophage polarization towards the M2 phenotype, which was partially achieved by the peroxisome proliferator-activated receptor γ (PPARγ)-mediated inhibition of NF-κB signaling. The PPARγ inhibitor GW9662 significantly attenuated the effects of ADM2. Besides, ADM2 treatment improved the AGE-impaired osteogenic potential of BMSCs in vitro. Furthermore, ADM2 accelerated bone regeneration, as revealed by improved radiological and histological manifestations and biomechanical parameters, accompanied by improved M2 macrophage polarization in diabetic DO rats, and these effects were partially blocked by GW9662 administration.

**Conclusions:**

These results indicate that ADM2 enhances diabetic bone regeneration during DO, by attenuating AGE-induced imbalances in macrophage polarization, partly through PPARγ/NF-κB signaling, and improving AGE-impaired osteogenic differentiation of BMSCs simultaneously. These findings reveal that ADM2 may serve as a potential bioactive factor for promoting bone regeneration under diabetic conditions, and imply that management of inflammation and osteogenesis, in parallel, may present a promising therapeutic strategy for diabetic patients during DO treatment.

**Supplementary Information:**

The online version contains supplementary material available at 10.1186/s13287-021-02368-9.

## Background

Distraction osteogenesis (DO) is widely accepted and applied in orthopedics and traumatology, because of its unique osteogenesis-inducing ability [1–3]. In the process of DO, gradual rhythmic traction is applied using an external fixator to fully induce neo-osteogenesis in the distraction zone [4]. However, bone regeneration is a complex physiological process regulated by multiple factors, and therefore, various metabolic disorders tend to impair bone regeneration during DO [5]. In clinical practice, diabetes mellitus (DM)-induced impairment of bone regeneration, characterized by a prolonged mineralization phase, is a relatively common condition, which leads to increased patient discomfort and complications [6–8]. As the number of people with DM is on the rise worldwide, with a predicted increase to a population of 592 million in 2035, there is a high demand for novel treatment strategies to accelerate bone regeneration in diabetic patients during DO [9].

DM-induced metabolic disorders exert detrimental effects on bone regeneration, leading to a greater risk of poor fracture healing or bone grafting failure [6, 10]. Several physiological conditions have been identified to contribute to DM-induced bone regeneration impairment, including insulin deficiency, accumulation of advanced glycation end products (AGEs), and elevated levels of circulatory homocysteine [11–13]. AGEs are formed by the non-enzymatic reaction of glucose with proteins under diabetic conditions and affect cellular functions upon interaction with its cell membrane-specific receptor [14, 15]. Previous studies have found that the interaction between AGEs and receptors of AGEs (RAGEs) could regulate various cellular signals, such as mitogen-activated protein kinase, hypoxia-induced factor-1α, peroxisome proliferator-activated receptor γ (PPARγ), and nuclear factor kappa-light-chain-enhancer of activated B cells (NF-κB), resulting in M1 macrophage polarization [16–19]. Meanwhile, prolonged inflammation mediated by M1 macrophages contributes to impaired osteogenic potential of bone marrow mesenchymal stem cell (BMSC) [20]. Additionally, AGEs have been shown to exert negative effects on stem cell osteogenic differentiation by modulating DNA methylation and the Wingless/Integrated (Wnt) signaling pathway [21]. Consequently, AGEs may be identified as an important therapeutic target to directly and indirectly attenuate BMSC dysfunction under diabetic conditions.

Adrenomedullin 2 (ADM2), also known as intermedin, is an endogenous peptide belonging to the calcitonin gene-related peptide (CGRP)/calcitonin family and is ubiquitously expressed in various tissues [22]. A previous study reported that systemic ADM2 levels were significantly decreased in diabetic rats compared to healthy individuals, indicating a relationship between low ADM2 levels and DM-related metabolic disorders [23]. ADM2 reportedly plays protective roles in the cardiovascular and renal systems via multiple mechanisms, such as anti-inflammation and inhibition of oxidative and endoplasmic reticulum stress [24–26]. Further, most peptides in the calcitonin family possess similar biological activities, and those of CGRP and ADM, which share the dimers of calcitonin receptor-like receptor (CLR) and receptor-modifying protein (RAMP) 1 or RAMP3 as common receptors with ADM2 [22], are of great significance for studying the role of ADM2 in macrophage polarization [27, 28]. Additionally, Pang et al. reported that ADM2 treatment may restore the M1/M2 balance and improve systemic insulin sensitivity in hyperhomocysteinemic mice [29]. Considering the increasing recognition of the vital role of M2 macrophage polarization in bone regeneration and decreased levels in DM [30], we speculated that ADM2 treatment may indirectly create a pro-regenerative microenvironment for enhanced bone regeneration under DM conditions, by facilitating a dynamic shift from M1 to M2 macrophage polarization. Moreover, the direct effect of ADM2 on the osteogenic differentiation of AGE-exposed BMSCs also remains largely unknown.

In this study, we investigated the roles of ADM2 in macrophage polarization and osteogenic differentiation of BMSCs under AGE exposure and explored the underlying mechanisms. Furthermore, a diabetic rat DO model was employed to examine the in vivo effects of ADM2 on bone regeneration and macrophage polarization.

## Methods

### Cell preparation and culture

Bone marrow-derived macrophages (BMDMs) and BMSCs were isolated from 4-week-old male C57BL/6 mice and male Sprague Dawley (SD) rats, respectively, by flushing the bone marrow from femurs and tibias with phosphate-buffered saline (PBS; HyClone, USA). BMDMs were cultured in Dulbecco’s modified Eagle’s medium (DMEM; HyClone, USA) supplemented with 10% (v/v) fetal bovine serum (FBS; Gibco, USA), 1% (v/v) penicillin-streptomycin (P/S; Gibco, USA), and 20% (v/v) L929 conditioned medium and identified by F4/80, a specific marker of murine macrophage populations, using flow cytometry. BMSCs were cultured in modified Eagle’s medium alpha (HyClone, USA) supplemented with 10% (v/v) FBS and 1% (v/v) P/S. L929 cells were cultured in DMEM supplemented with 10% (v/v) FBS and 1% (v/v) P/S. Cells should become confluent in 2 to 3 days, and the supernatant medium was collected 3 days later. The conditional medium was filtered (0.22 μm) and stored at −80 °C. All cells were cultured at 37 °C in a humidified atmosphere with 5% CO_2_.

### Macrophage treatment

BMDMs were stimulated with AGEs (200 μg/ml; BioVision, USA) for 48 h in the presence or absence of ADM2 (1 μM; Phoenix Pharmaceuticals, USA). An equal volume of PBS was added to the control group. In addition, GW9662 (2 μM, pretreatment for 2 h; Beyotime, China) was administrated along with AGEs and ADM2 treatment to verify the molecular mechanism by which ADM2 regulates AGE-induced macrophages.

### Flow cytometry analysis

After treatment, BMDMs were fixed with 4% (w/v) paraformaldehyde (PFA), blocked with 5% (w/v) bovine serum albumin (BSA), and then incubated with FITC-conjugated F4/80 antibody (11-4801-82, eBioscience, USA), APC-conjugated CD206 antibody (17-2061-82, eBioscience, USA), and PE-conjugated CD86 antibody (12-0862-82, eBioscience, USA) for 30 min. The candidate cells were detected using a BD FACS Caliber flow cytometer and analyzed using FlowJo v10.0 software. F4/80^+^ cells were identified as macrophages (Figure S1), and the expression levels of CD86 and CD206 were detected to evaluate the M1 and M2 polarization states of BMDMs.

### Enzyme-linked immunosorbent assay (ELISA)

The media supernatant was collected from cultured BMDMs and stored at −80 °C. The concentrations of bone morphogenetic protein 2 (BMP-2), insulin-like growth factor 1 (IGF-1), tumor necrosis factor α (TNF-α), and transforming growth factor β (TGF-β) were determined using ELISA kits (E04509m, E04581m, E04744m, E04726m, CUSABIO, China), according to the manufacturer’s protocols.

### Immunofluorescence staining

BMDMs were fixed with 4% (w/v) PFA, washed with PBS thrice, blocked with 5% (w/v) BSA for 1 h, and then incubated with the primary antibody against p65 (1:100; #8242, Cell Signaling Technology, USA) at 4 °C overnight. The cells were then incubated with the Cy3-conjugated secondary antibody (1:1000; ab6939, Abcam, UK) at 25 °C for 1 h and then stained with 4′,6-diamidino-2-phenylindole (DAPI) for 5 min. The activation and nuclear translocation of p65 were observed using a fluorescence microscope.

### Osteogenic differentiation and detection

To determine the effects of ADM2 on the osteogenic differentiation of AGE-induced BMSCs, both alkaline phosphatase (ALP) and mineral deposition were detected. Briefly, BMSCs were inoculated in 24-well plates (5×10^4^/well). At 80% confluence, the medium was replaced with osteogenic induction medium (OIM; 20 mM β-glycerophosphate, 1 nM dexamethasone, and 50 μM L-ascorbic acid-2-phosphate in the complete medium; Sigma-Aldrich, USA) containing AGEs (200 μg/ml) in the presence or absence of ADM2 (1 μM), and the medium was replenished every 2 days. ALP staining and activity assays were performed 7 days after osteogenic induction according to the manufacturer’s instructions (Beyotime, China). On the 14th day of differentiation, alizarin red S (ARS; Cyagen Biosciences, China) staining was performed to evaluate mineral deposition. For quantitative analysis of mineralization, calcium deposition was eluted with 10% (w/v) cetylpyridinium chloride (Sigma-Aldrich, USA), and the OD value was measured at 570 nm.

### Quantitative real-time polymerase chain reaction (qRT-PCR) analysis

Total RNA was extracted using an RNA Purification Kit (EZBioscience, USA) and cDNA was obtained from 500 ng of total RNA using the Reverse Transcription Kit (EZBioscience, USA). qRT-PCR was then performed using SYBR Green qPCR Master Mix (EZBioscience, USA). Relative gene expression levels were calculated using the 2^−△△CT^ method and GAPDH was used as the reference gene for normalization. The primer sequences are shown in Table 1.

**Table 1:** Primers for quantitative real-time polymerase chain reaction (qRT-PCR)

Gene	Forward (5′–3′)	Reverse (5′–3′)
ALP	CCGCAGGATGTGAACTACT	GGTACTGACGGAAGAAGGG
Arg-1	CAGAAGAATGGAAGAGTCAG	CAGATATGCAGGGAGTCACC
IL-6	AGCCAGAGTCCTTCAGAGAGAT	GCACTAGGTTTGCCGAGTAGAT
iNOS	CGAGACGGATAGGCAGAGATTG	CTCTTCAAGCACCTCCAGGAA
MRC1	CCTATGAAAATTGGGCTTACGG	CTGACAAATCCAGTTGTTGAGG
OCN	CAGACAAGTCCCACACAGCA	CCAGCAGAGTGAGCAGAGAGA
OPN	GGCCGAGGTGATAGCTT	CTCTTCATGCGGGAGGT
OSX	GGAAAAGGAGGCACAAAGAA	CAGGGGAGAGGAGTCCATT
TGF-β	CGGAGAGCCCTGGATACCA	GCCGCACACAGCAGTTCTT
TNF-α	GCTGAGCTCAAACCCTGGTA	CGGACTCCGCAAAGTCTAAG
GAPDH (mouse)	AAATGGTGAAGGTCGGTGTG	AGGTCAATGAAGGGGTCGTT
GAPDH (rat)	ATGGCTACAGCAACAGGGT	TTATGGGGTCTGGGATGG

### Western blot analysis

Total protein was extracted using RIPA lysis buffer with protease and phosphatase inhibitors (Solarbio, China) at 4 °C. Protein concentration was determined using a BCA Protein Assay Kit (EpiZyme, China). Equal amounts of protein (30 μg) were subjected to 10% (w/v) SDS-PAGE and then transferred to a polyvinylidene difluoride membrane (Millipore, USA). After blocking with 5% (w/v) BSA, the membrane was incubated with primary antibodies at 4 °C overnight and then incubated with horseradish peroxidase (HRP)-conjugated secondary antibodies (1:10000; 115-035-003, 111-035-003, Jackson ImmunoResearch, USA) at 25 °C for 1 h. Immunoreactive bands were visualized using enhanced chemiluminescence reagent (Millipore, USA) and the grayscale of protein bands were semi-quantified using ImageJ software.

The primary antibodies used in this study included anti-PPARγ (1:1500; #2435, Cell Signaling Technology, USA), anti-IκBα (1:1500; #4814, Cell Signaling Technology, USA), anti-p65 (1:1500; #8242, Cell Signaling Technology, USA), anti-phosphorylated p65 (p-p65; 1:1500; #3033, Cell Signaling Technology, USA), anti-BMP-2 (1:1500; ab214821, Abcam, UK), anti-OSX (1:1500; ab209484, Abcam, UK), anti-OCN (1:1000; A6205, ABclonal, China), and anti-GAPDH (1:2000; #5174, Cell Signaling Technology, USA).

### Induction of the type 1 diabetes mellitus (T1DM) rat model

All experimental procedures were approved by the Animal Research Committee of Shanghai Jiao Tong University Affiliated Sixth People’s Hospital. After fasting for 12 h, a single high dose (65 mg/kg) of streptozotocin (STZ; 10 mg/ml in 0.01 M citrate buffer; Sigma-Aldrich, USA) was intraperitoneally injected into SD rats, weighing 350–400 g, to establish T1DM models. After STZ injection of 7 days, random plasma glucose levels (PGLs) were determined using a glucometer and blood from the tail vein. Rats with PGLs above 16.7 mmol/l were considered as diabetic individuals, and those that failed to reach the target glycemic index were excluded from the study.

### Animal surgery and treatment

All experimental procedures were approved by the Animal Research Committee of Shanghai Jiao Tong University Affiliated Sixth People’s Hospital (DWSY2019-0172). A total of 36 T1DM SD rats were used in this study and randomly assigned to the DM (*n* = 12), DM+ADM2 (*n* = 12), and DM+ADM2+GW9662 (*n* = 12) groups. Rats injected with an equal volume of citrate buffer were assigned to the non-diabetic control group (*n* = 12). To establish the DO model, a transverse osteotomy was performed at the midshaft of the right tibia after anesthesia and exposure. Next, a specially designed monoliteral external fixator (Xinzhong Company, China) was mounted to fix the proximal and distal segments of the tibia. Thereafter, surgical incisions were closed layer-wise. The periosteum was preserved as much as possible during the procedure. The DO procedures comprised three phases: latency phase for 5 days, distraction phase for 10 days (0.25 mm every 12 h), and consolidation phase for 4 weeks. ADM2 (200 μg/kg/day) was subcutaneously injected during the consolidation phase into the DM+ADM2 group and DM+ADM2+GW9662 group, and the latter was intraperitoneally administrated GW9662 (1 mg/kg/day). Equal-volume PBS was subcutaneously injected at the same time as the DM and control groups.

### Digital radiography and micro-computed tomography (CT)

X-ray films, which were focused on the distraction gaps, were acquired weekly from the beginning of the consolidation phase. The lengthened tibia specimens were harvested 2 (*n* = 6) and 4 (*n* = 6) weeks after distraction. Micro-CT scans (Skyscan 1172, Bruker, Germany), with a voltage of 80 kV, a current of 112 μA, and an exposure time of 370 ms, were performed to quantitatively evaluate bone regeneration in the distraction zone. Three-dimensional (3D) reconstructions of the regenerated callus were produced using the CTVox software. Parameters including bone mineral density (BMD) and bone volume/tissue volume (BV/TV) of the regenerated bone were analyzed using the CTAn software.

### Biomechanical testing

The mechanical characteristics of the fresh tibia specimens (*n* = 3) were determined using a four-point bending device after 4-week consolidation. During the test, the tibia specimens were loaded in the anterior-posterior direction with the posterior side in tension. The modulus of elasticity (E-modulus), ultimate load, and energy to failure were recorded and analyzed using Vernier Graphical Analysis software.

### Histological and immunohistochemical staining

For histological analyses, after 2 (*n* = 3) and 4 (*n* = 3) weeks of consolidation, tibia specimens were fixed in 4% (w/v) PFA for 24 h, decalcified in 10% (w/v) ethylene diamine tetraacetic acid (EDTA; pH = 7.4) for 21 days, dehydrated using graded ethanol of increasing concentrations, and then embedded in paraffin. Samples were cut into 5-μm-thick longitudinally oriented sections and then subjected to hematoxylin-eosin (H&E), Masson’s trichrome, and Safranin O-Fast Green (SO-FG) staining.

For immunohistochemical staining, sections were incubated in 0.3% (v/v) hydrogen peroxide for 20 min to quench endogenous peroxidase activity. After antigen retrieval in 0.01 mol/l citrate buffer (pH 6.0) at 65 °C for 20 min and blocking with 5% (v/v) goat serum for 1 h, sections were incubated with anti-OCN antibody (1:100; A6205, ABclonal, China) at 4 °C overnight. After incubation with secondary antibodies conjugated with HRP (1:1000; 111-035-003, Jackson ImmunoResearch, USA) at 25 °C for 1 h, an HRP-streptavidin system was used to detect positive areas followed by counterstaining with hematoxylin.

### Immunofluorescent analysis

CD68 and CD86 or CD68 and CD206 double immunofluorescence staining was performed to detect M1 or M2 macrophages, respectively. After consolidation for 2 weeks, tibia specimens (*n* = 3) were decalcified in 18% (w/v) EDTA for 3 days after fixation. Subsequently, the samples were dehydrated in 30% (w/v) sucrose, embedded in optimal cutting temperature compound, and cut into 10-μm-thick longitudinally oriented sections. After blocking with 5% (w/v) BSA for 1 h, bone sections were incubated with primary antibodies overnight at 4 °C, followed by incubation with fluorophore-conjugated secondary antibodies (1:200; ab6785, ab6939, Abcam, UK) at 25 °C for 1 h. Nuclei were stained with DAPI. A fluorescent microscope was used for observation and image capture. For semiquantitative analysis, the ratios of CD86^+^CD68^+^/CD68^+^ cells and CD206^+^CD68^+^/CD68^+^ cells in the distraction area were calculated using Image-Pro Plus software.

The primary antibodies used in this study included anti-CD68 (1:100; ab125212, Abcam, UK), anti-CD86 (1:100; IMG-6882A, Novus, USA), and anti-CD-206 (1:100; ab8918, Abcam, UK).

### Statistical analysis

All data are presented as mean ± standard deviation. The statistical differences were analyzed using one-way analysis of variance (ANOVA) followed by Tukey’s post hoc test among groups using GraphPad Prism 8 software. Results were considered statistically significant at a two-tailed *P*-value less than 0.05.

## Results

### ADM2 reversed AGE-induced M1 macrophage polarization to the M2 phenotype

**Fig. 1:**
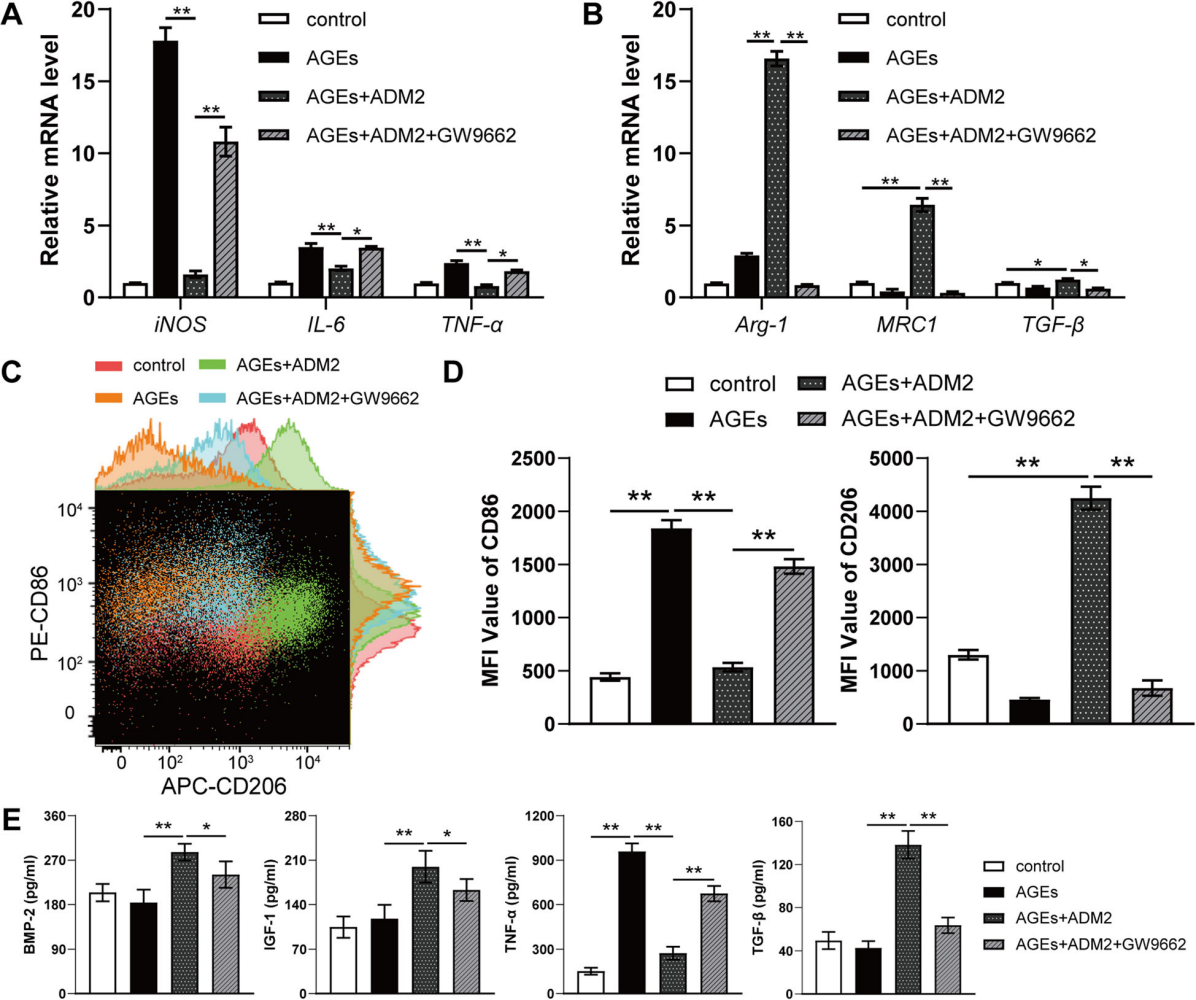
ADM2 reversed AGE-induced M1 macrophage polarization to M2 phenotype. **A**, **B** Expression of M1-specific genes (*iNOS*, *IL-6*, and *TNF-α*) (**A**) and M2-specific genes (*Arg-1*, *MRC1*, and *TGF-β*) (**B**) of BMDMs treated with vehicle, AGEs, AGEs+ADM2, and AGEs+ADM2+GW9662 was assessed using qRT-PCR. **C** The expression of CD86 (M1 surface marker) and CD206 (M2 surface marker) on BMDMs in each group was examined using flow cytometry. **D** Quantification of mean fluorescence intensity (MFI) of the surface markers. **E** ELISA for production of pro-inflammatory cytokine (TNF-α) and anti-inflammatory, osteogenic cytokines (BMP-2, IGF-1, and TGF-β) in the supernatant of BMDMs in each group. The data were confirmed by one-way analysis of variance (ANOVA) followed by Tukey’s post hoc test from three independently repeated tests and are presented as the means ± SD. *P < 0.05; **P < 0.01

AGE exposure significantly promoted the expression of genes related to M1 polarization, including *iNOS*, *IL-6*, and *TNF-α* (Fig. 1A). However, ADM2 administration reversed the AGE-induced elevation of M1 marker gene expression and further promoted the expression of genes related to M2 polarization, including *Arg-1*, *MRC1*, and *TGF-β* (Fig. 1A, B). Flow cytometry results showed a similar trend: AGE exposure amplified the population of M1 macrophages, while ADM2 administration reversed M1 polarization to the M2 phenotype (Fig. 1C, D). Additionally, ELISA results revealed that AGEs significantly promoted the secretion of pro-inflammatory cytokine TNF-α in BMDMs, while ADM2 not only moderated the pro-inflammatory effect of AGEs but also increased the production of anti-inflammatory and osteogenic cytokines, including BMP-2, IGF-1, and TGF-β (Fig. 1E).

### ADM2 attenuated AGE-induced activation of NF-κB through the PPARγ/IκBα pathway

**Fig. 2:**
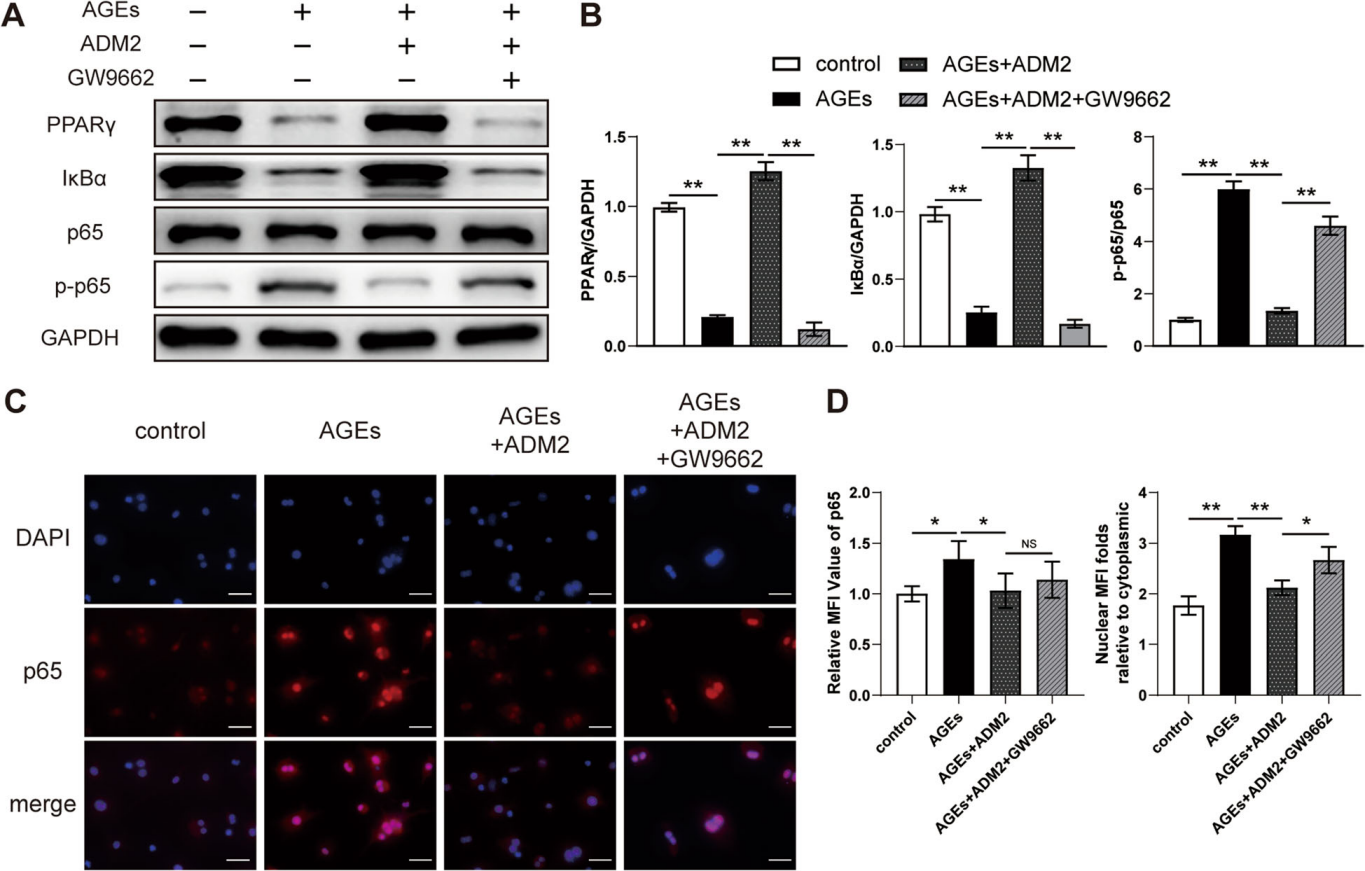
ADM2 reversed the imbalance of M1/M2 polarization induced by AGEs via the PPARγ/IκBα/NF-κB pathway. **A** Western blot of PPARγ, IκBα, p65, and p-p65 in BMDMs treated with vehicle, AGEs, AGEs+ADM2, and AGEs+ADM2+GW9662. **B** Quantitative analysis of PPARγ and IκBα protein levels relative to GAPDH and the phosphorylated level of p65. **C** Representative immunocytochemistry images showing the expression and distribution of p65 in BMDMs in each group. Scale bar 50 μm. **D** Quantitative analysis of the expression and nuclear translocation of p65. The data were confirmed by one-way analysis of variance (ANOVA) followed by Tukey’s post hoc test from three independently repeated tests and are presented as the means ± SD. *P < 0.05; **P < 0.01

The NF-κB pathway plays an essential role in M1 macrophage polarization, and PPARγ modulates NF-κB-dependent inflammation by upregulating the expression of IκBα, a negative regulator of p65 [31]. As shown by western blotting, the protein expression of total PPARγ and IκBα was significantly suppressed by AGEs, leading to NF-κB p65 activation (Fig. 2A, B), which was also confirmed using immunofluorescence staining for p65 expression and nuclear translocation (Fig. 2C, D). However, ADM2 treatment rescued the expression of PPARγ and IκBα and subsequently diminished the activation and nuclear translocation of p65 (Fig. 2A–D). When BMDMs were treated with the PPARγ antagonist GW9662 along with AGEs and ADM2, the effects of ADM2 on the PPARγ/IκBα/NF-κB pathway were partially abated (Fig. 2A–D), leading to enhanced M1 polarization with respect to gene expression, surface marker expression, and cytokine production (Fig. 1A–E). Therefore, these findings indicate that ADM2 reversed AGE-induced macrophage inflammation via the PPARγ/IκBα/NF-κB pathway.

### ADM2 rescued AGE-mediated impairment of osteogenic potential

**Fig. 3:**
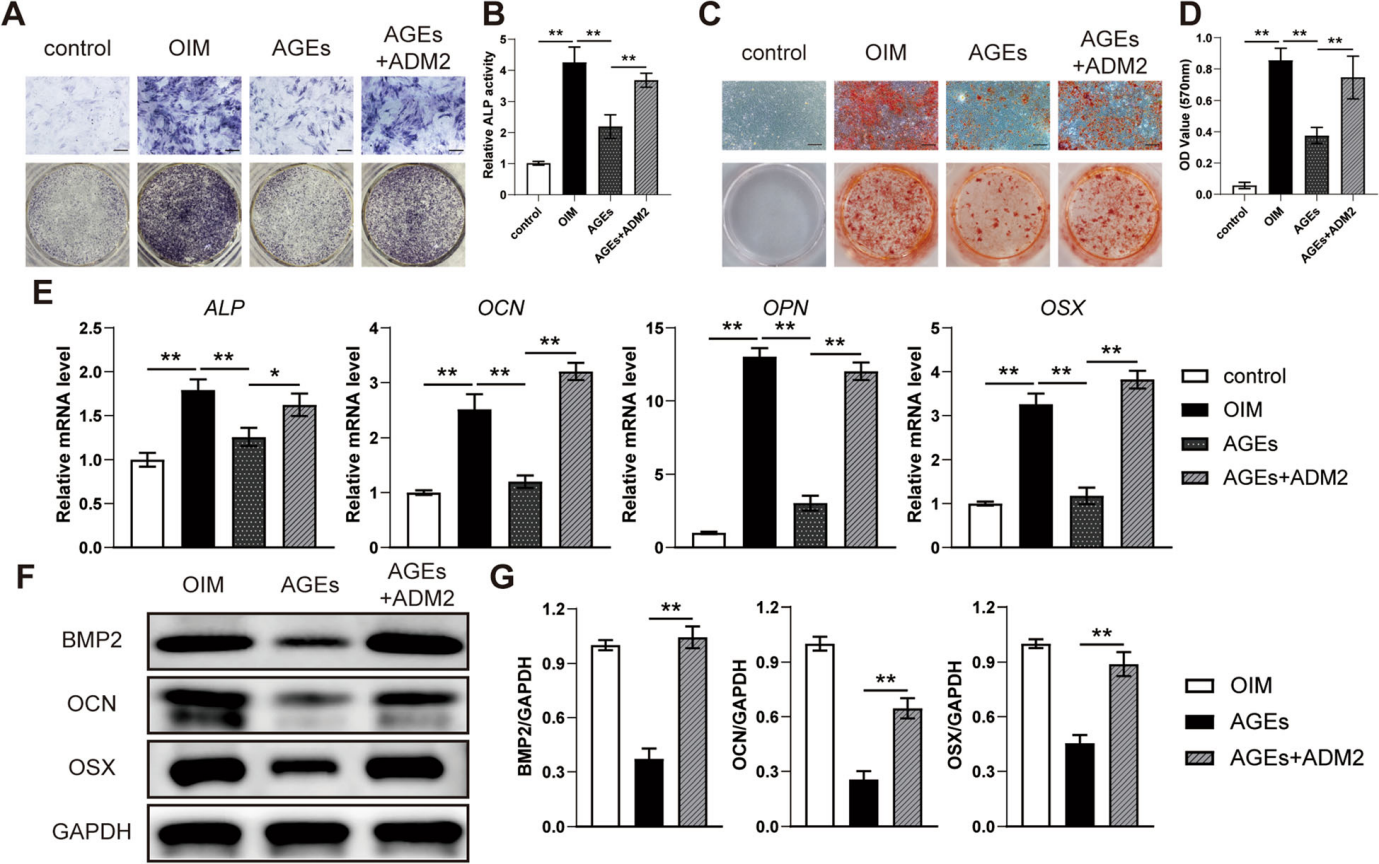
ADM2 improved the AGE-impaired osteogenic potential of BMSCs. **A**–**D** Osteogenesis of BMSCs treated with α-MEM, OIM, OIM+AGEs, and OIM+AGEs+ADM2 was determined using ALP staining (**A**), ALP activity assays (**B**), and alizarin red staining (**C**). Calcium deposition was assessed by measuring the optical density (**D**). Scale bar 200 μm. **E**, **F** Expression of osteogenic-specific genes and proteins of BMSCs in different groups were assessed using qRT-PCR (**E**) and western blot (**F**). **G** Quantitative analysis of the protein levels of BMP-2, OCN, and OSX relative to GAPDH. The data were confirmed by one-way analysis of variance (ANOVA) followed by Tukey’s post hoc test from three independently repeated tests and are presented as the means ± SD. *P < 0.05; **P < 0.01

To investigate the effect of AGEs and the protective potential of ADM2 on the osteogenesis of BMSCs in vitro, ALP staining, ALP activity, and ARS staining were performed. As evidenced by the qualitative and quantitative results, AGE-induced impairments of ALP activity and matrix mineralization were attenuated by ADM2 treatment (Fig. 3A–D). Moreover, we observed that osteogenic genes, including *ALP*, *OCN*, *OPN*, and *OSX*, were significantly upregulated after ADM2 treatment (Fig. 3E). Western blotting revealed that ADM2 treatment upregulated expression levels of BMP-2, OCN, and OSX in BMSCs under AGE exposure (Fig. 3F, G), suggesting that exogenous ADM2 administration partially rescued the osteogenic potential of BMSCs impaired by AGEs.

### ADM2 accelerated bone formation and consolidation during DO in diabetic rats

**Fig. 4:**
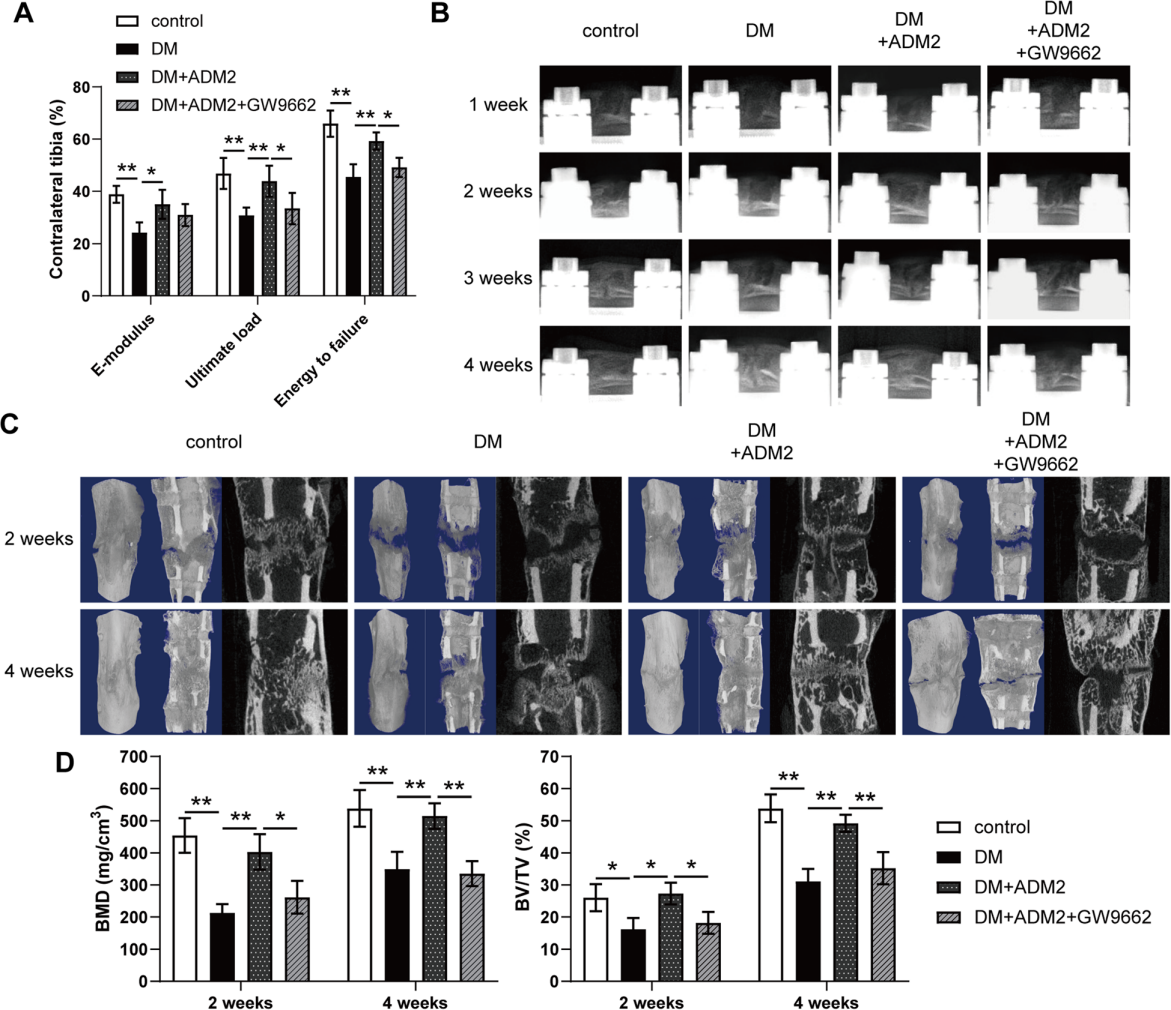
ADM2 accelerated bone consolidation during distraction osteogenesis in diabetic rats. **A** Mechanical tests (E-modulus, ultimate load, and energy to failure) of the distracted tibias. The values were normalized to the corresponding contralateral normal tibias. **B** Representative X-rays of distraction regenerate at various time points of the consolidation phase in the four groups. **C** Representative 3D and longitudinal images of the tibial distraction zone after 2 and 4 weeks of consolidation and treatment. **D** Quantitative analysis of bone mineral density (BMD) and bone volume/tissue volume (BV/TV) of the regenerated bone. The data were confirmed by one-way analysis of variance (ANOVA) followed by Tukey’s post hoc test from three independently repeated tests and are presented as the means ± SD. *P < 0.05; **P < 0.01

As shown in Fig. 4A, detection of mechanical properties of fresh tibia specimens exhibited improved ultimate load, energy to failure, and elasticity modulus in the DM+ADM2 group and jeopardized parameters in the DM+ADM2+GW9662 group (Fig. 4A). Besides, a series of representative X-ray images across the DO time course showed the progression of bone consolidation (Fig. 4B). Opaque callus appearing in the distraction regenerates was jeopardized in the DM group and rescued by ADM2 treatment in terms of volume and continuity of the callus in the middle of the consolidation phase. However, recovery of bone regeneration was compromised by GW9662 administration. At the end of the consolidation phase, the cortical bone within the distraction area is nearly continuous with abundant callus in the control and DM+ADM2 groups. Nevertheless, the bone regeneration in the DM and DM+ADM2+GW9662 groups remained unsatisfactory, with a certain amount of neo-callus and discontinuous cortical bone. Similar observations were confirmed by micro-CT examination of distraction regenerates at 2 and 4 weeks after the distraction phase (Fig. 4C). The BMD and BV/TV in the distraction gaps were impaired in the DM group and improved by ADM2 treatment (Fig. 4D). However, GW9662 co-administration partially compromised the protective effect of ADM2 (Fig. 4D), indicating that ADM2 induced preferable bone regeneration in a diabetic DO model, at least partially, through PPARγ activation.

### ADM2 administration accelerated mineralized callus formation within the distraction zone

**Fig. 5:**
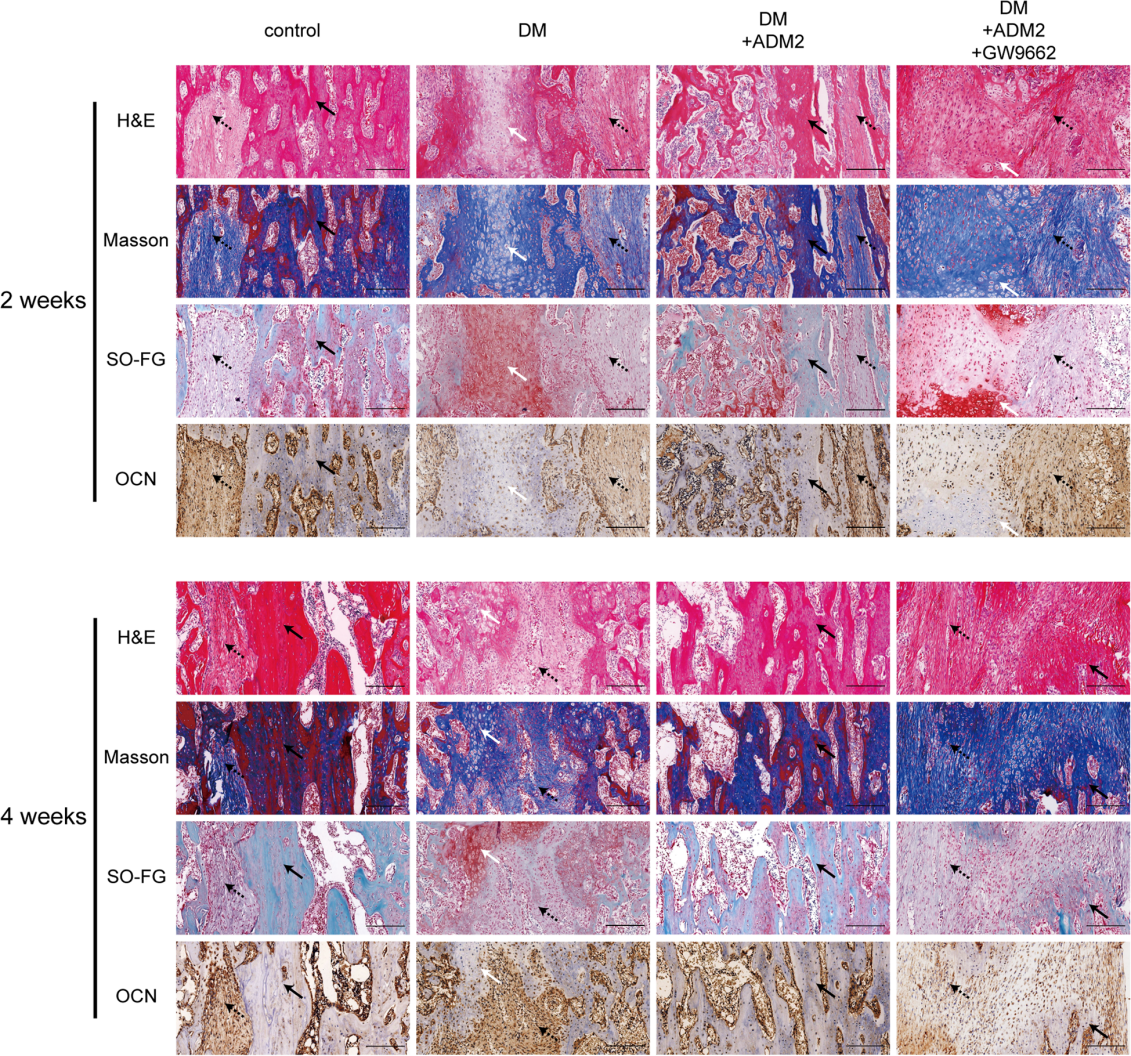
ADM2 accelerated mature callus formation during distraction osteogenesis in diabetic rats. Representative images of H&E, Masson, and Safranin O-Fast Green staining from the middle of the distracted tibias showed various amounts of trabecular bones, fibrous tissue, and cartilaginous tissue in all four groups. Compared to the control group, the distraction gaps showed increased fibrous and cartilaginous tissues and less trabecular bone in the center zone in the DM group. Upon treatment of ADM2, the distraction gaps showed more mature trabecular bone and less fibrous and cartilaginous tissues. The bone-regenerating effect of ADM2 was partially reversed by GW9662 administration. Immunohistochemical analysis for osteocalcin (OCN) showed a similar tendency with stronger staining in the control and ADM2 groups than that in the DM and GW9662 groups. Dotted arrows, fibrous-like tissue. White arrows, cartilaginous tissue. Black arrows, newly formed trabecular bone. Scale bar 200 μm

As shown in Fig. 5, H&E, Masson’s trichrome, and SO-FG staining of the distraction regenerates revealed various amounts of newly formed trabecular bone, cartilaginous tissue, and fibrous-like tissue, parallel with the distraction forces (Fig. 5). Distraction regenerates treated with ADM2 exhibited enhanced bone consolidation at 2 and 4 weeks after distraction in comparison with the DM group, which was evidenced by higher levels of mature trabecular bone and lower levels of fibrous-like tissue in the ADM2 group (Fig. 5). However, the ossification process was impeded by GW9662 (Fig. 5). In addition, immunohistochemical analysis of distraction regenerates revealed a similar tendency: after 2 weeks of consolidation, a higher expression of OCN, especially around the neo-formed trabecular bone, was confirmed within the distraction areas of the control and DM+ADM2 groups, demonstrating the active osteogenic process of ADM2-treated rats in the middle of the consolidation phase (Fig. 5). Thereafter, the expression of OCN in the DM and DM+ADM2+GW9662 groups was gradually elevated, indicating that osteogenesis was initially processed in the distraction zone of these groups, although 2 weeks later than the control and DM+ADM2 groups (Fig. 5).

### ADM2 restored the imbalance of macrophage polarization during DO in diabetic rats

**Fig. 6:**
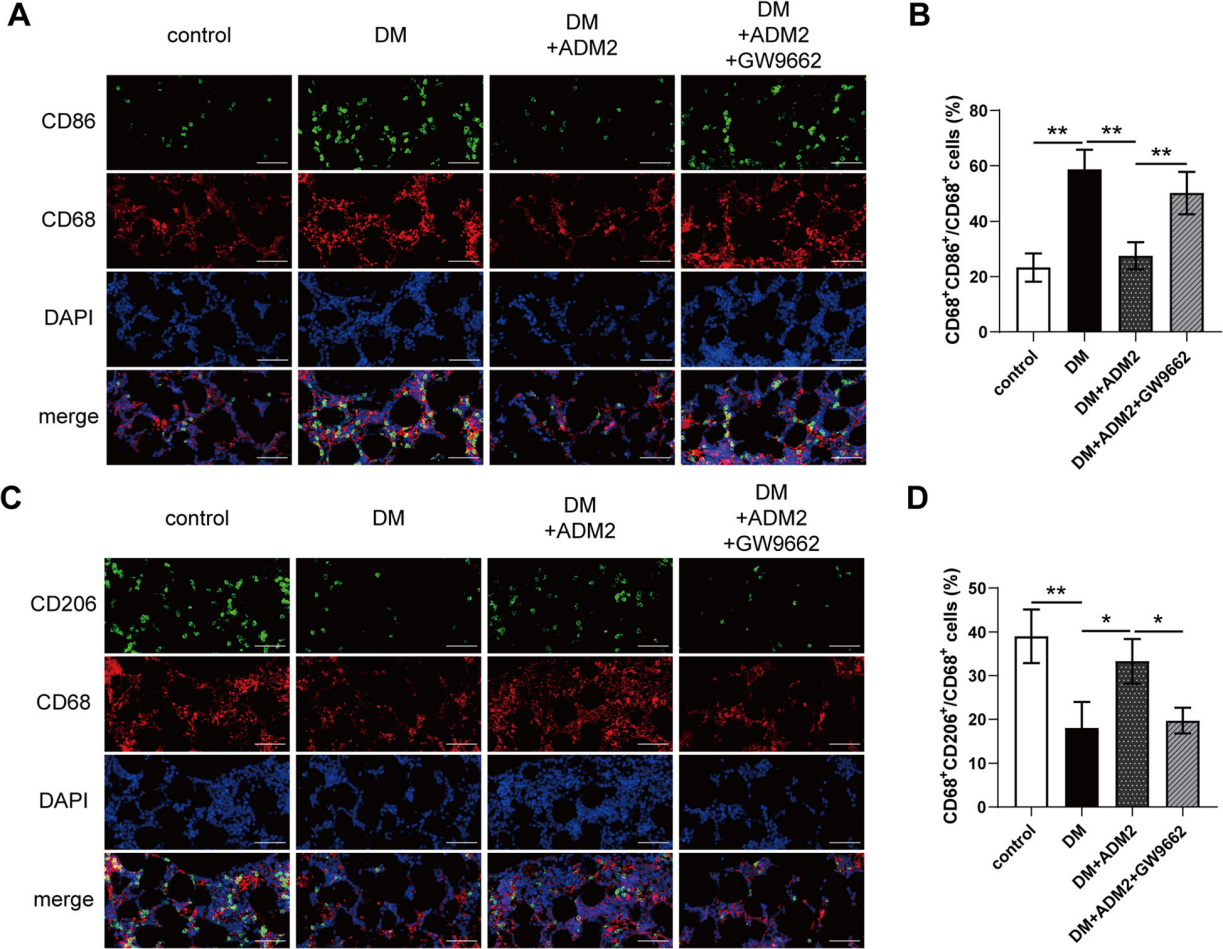
ADM2 reversed the imbalance of M1/M2 polarization during distraction osteogenesis in diabetic rats. **A** Immunofluorescence staining images of CD68 and CD86 for the distraction area sections from the four groups. Scale bar 100 μm. **B** Quantitative analysis of CD68^+^CD86^+^/CD68^+^ M1 macrophage ratio for the distraction area sections from the four groups. **C** Immunofluorescence staining images of CD68 and CD206 for the distraction area sections from the four groups. Scale bar 100 μm. **D** Quantitative analysis of CD68^+^CD206^+^/CD68^+^ M2 macrophage ratio for the distraction area sections from the four groups. The data were confirmed by one-way analysis of variance (ANOVA) followed by Tukey’s post hoc test from three independently repeated tests and are presented as the means ± SD. *P < 0.05; **P < 0.01

To explore whether ADM2 promoted M2 polarization within distraction regenerates, we applied double immunofluorescence to label M1 (CD68^+^CD86^+^) and M2 (CD68^+^CD206^+^) macrophages. As shown in Fig. 6, there was minimal detection of M1 macrophages in the control group, but enriched distribution in the DM group (Fig. 6A, B). However, after ADM2 treatment, the ratio of M1 macrophages significantly decreased (Fig. 6A, B). Conversely, the ratio of M2 macrophages was minimal in the DM group, but increased in the DM+ADM2 group (Fig. 6C, D). Moreover, these effects of ADM2 were mostly compromised by GW9662 administration (Fig. 6A–D). Taken together, these results indicate that ADM2 induced macrophage M2 polarization from the M1 phenotype, in distraction regenerates of diabetic DO rats.

## Discussion

In this study, we found that ADM2 reversed AGE-induced M1 macrophage polarization to M2 phenotype in vitro. In addition, the M2 polarization effect of ADM2 was achieved, at least in part, by the inhibition of NF-κB signaling via the activation of PPARγ. Moreover, we verified the rescue effect of ADM2 on AGE-induced BMSC dysfunction during osteogenic differentiation. In vivo, the rescue effects of ADM2 on bone regeneration and M2 macrophage polarization under DM were verified and the involvement of PPARγ activation in these effects of ADM2 was also investigated. To the best of our knowledge, this is the first study to show that ADM2 can accelerate bone regeneration under diabetic conditions by regulating macrophage polarization and osteogenesis in parallel.

**Fig. 7:**
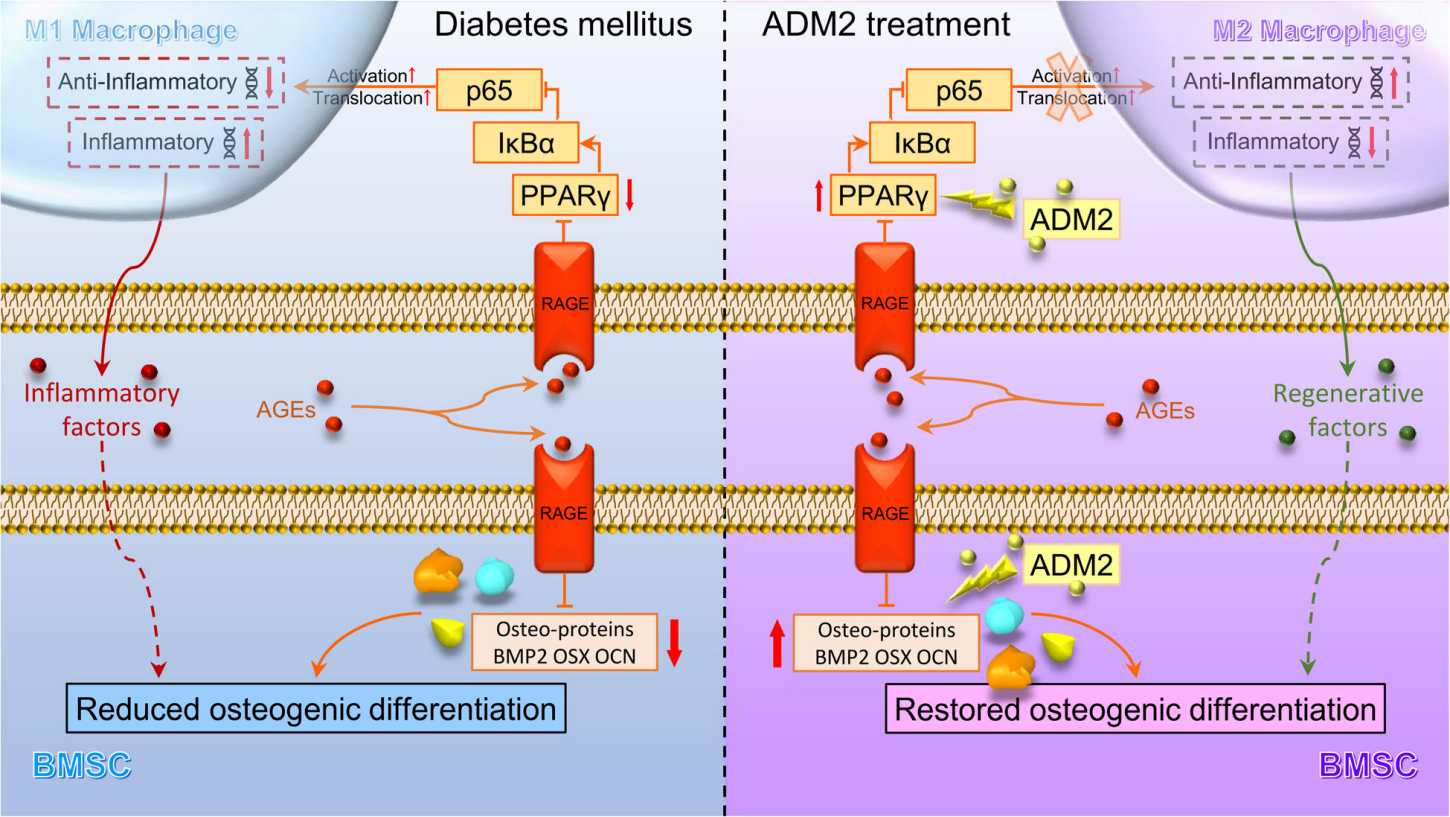
Working model of ADM2 restoration of AGE-induced imbalanced macrophage polarization and impaired osteogenesis. Under pathological diabetic conditions, AGEs interact with RAGE in macrophages and BMSCs, activating AGE-RAGE signaling. In macrophages, PPARγ is downregulated, leading to inhibition of IκBα and activation and nuclear translocation of p65, which plays a vital role in the M1 polarization of macrophages. The inflammatory cytokines secreted by M1 macrophages would lead to an inflammatory microenvironment, thus indirectly impairing the osteogenic differentiation of BMSCs. Besides, the activation of AGE-RAGE signaling in BMSCs could directly impair the osteogenic potential of BMSCs. Finally, reduced osteogenesis of BMSCs causes impaired bone regeneration in T1DM. ADM2 reverses AGE-induced M1 polarization of macrophages to M2 phenotype, which contributes to the regenerative microenvironment, by activating PPARγ, and attenuates AGE-impaired osteogenic potential of BMSCs simultaneously, thus accelerating diabetic bone regeneration during DO

Even with insulin replacement therapy, a high rate of prolonged consolidation is observed in most T1DM patients undergoing DO treatment, and this effect is primarily attributed to impaired bone regeneration [8, 32]. Considerable evidence indicates that BMSCs deteriorate under diabetic conditions and exhibit reduced osteogenic capability [21, 33, 34]. Although the specific mechanism of diabetes-induced BMSC dysfunction is not fully understood, the AGE/RAGE pathway is considered as one of the primary mechanisms. On the one hand, AGEs have been reported to directly interact with RAGEs of osteoblast lineage cells and impair osteogenic differentiation by modulating DNA methylation and Wnt signaling [21]. On the other hand, as knowledge regarding cellular mechanisms underlying bone regeneration in DM expands, recent studies acknowledged that osteogenic differentiation of BMSCs is, to a great extent, suppressed by prolonged inflammation under diabetic conditions [35, 36]. Since a pathologically elevated ratio of M1 macrophages is the fundamental cause of prolonged inflammation [37], AGE-induced M1 macrophage polarization may serve as another promising treatment candidate for diabetic bone regeneration in DO [38]. Therefore, the present study established the simultaneous attenuation of AGE-induced M1 polarization and BMSC dysfunction, which intervenes in the indirect and direct factors leading to impaired osteogenesis, as a therapeutic strategy, and verified that ADM2 could indeed improve bone regeneration under diabetic conditions by exerting this dual positive effect (Fig. 7).

The NF-κB family of transcription factors plays an essential role in inflammation and macrophage M1 polarization induced by various molecules, including AGEs [39]. Although much research has been conducted on the NF-κB-inhibiting effect of ADM2, the underlying mechanism remains unclear [23, 40, 41]. PPARγ is a key nuclear transcription factor involved in inflammation and macrophage polarization [42, 43]. A recent study verified that PPARγ stimulation could inhibit the activation of NF-κB through upregulation of IκBα expression at the transcriptional level, which retains the NF-κB subunits p50/p65 in a cytoplasmic inactive complex [31]. Of note, ADM2 reportedly exhibits anti-inflammatory and M2 polarization effects; therefore, we hypothesized that ADM2 may exert a positive effect on PPARγ activation in BMDMs. Indeed, we observed that the ADM2 treatment significantly rescued the expression of PPARγ and IκBα, which was downregulated by AGEs. As expected, the activation and nuclear translocation of NF-κB were also diminished during ADM2-induced M2 macrophage polarization. In the diabetic DO model, ADM2 administration distinctly increased the ratio of M2 macrophages within distraction regenerates. Moreover, the M2 polarization effect of ADM2 in vitro and in vivo could be at least partially reversed by a PPARγ antagonist, indicating that ADM2 might facilitate the dynamic shift from M1 to M2 phenotype under diabetic conditions through the PPARγ/IκBα/NF-κB pathway. However, various pathways participate in AGE-induced M1 macrophage polarization under DM conditions, and the comprehensive mechanisms, except for PPARγ activation, induced by ADM2 are poorly understood [44–46]. Since CGRP reportedly promotes M2 macrophage polarization [28] and ADM could also activate PPARγ signaling [47], we speculate that the biological effects of ADM2 in the present study may be directly mediated through its interaction between CLR/RAMP1 and CLR/RAMP3, which are the common receptors of ADM2 with CGRP and ADM [22], although this hypothesis requires further verification. In addition, in order to inhibit the pathophysiological process caused by AGE/RAGE interaction, direct upstream interventions also represent a potential therapeutic strategy, including inhibition of AGE formation, downregulation of RAGE expression, and blockage of AGE/RAGE interaction [48–50]. Therefore, other feasible mechanisms by which ADM2 accelerates diabetic bone regeneration are yet to be explored.

Although the osteogenic differentiation process was, to a great extent, suppressed by prolonged inflammation under diabetic conditions, AGE could also directly inhibit the osteogenic potential of adipose-derived stem cells [21]. Hence, although prolonged inflammation under diabetic conditions could be relieved by ADM2 treatment, the AGE-induced direct osteogenesis impairment still needs to be retrieved for full restoration of bone regeneration under diabetic conditions. In this study, we verified that ADM2 partially rescued the AGE-impaired osteogenic capacity of BMSCs. This discovery, along with the promotive effect of ADM2 on M2 polarization, provided theoretical support for the notion that ADM2 facilitates diabetic bone regeneration during DO. Based on previous studies, the inhibitory effect of AGEs on osteogenic differentiation is closely related to DNA methylation and downregulation of the Wnt pathway [21]. Since ADM2 has been shown to activate protein kinase B (AKT) signaling in various cells [51–53], and activated AKT could preserve β-catenin through phosphorylation and inactivation of glycogen synthase kinase-3 β (GSK-3β) [54], we assume that the osteogenesis-protective effect of ADM2 on BMSCs may contribute to the activation of the AKT/GSK-3β/β-catenin pathway. Although this study does not include an exploration of the mechanisms by which ADM2 directly improves the osteogenic potential of BMSCs, further studies are required to help develop a comprehensive and in-depth understanding of the relevance of ADM2 with bone regeneration under diabetic conditions.

The present study has several limitations. First, the detailed mechanisms underlying the ability of ADM2 to activate PPARγ remain to be fully elucidated. Second, the feasible mechanisms contributing to the rescue effect of ADM2 on AGE-impaired osteogenic potential have not been verified. Lastly, although ADM2 could rescue the osteogenic potential of BMSCs impaired by AGEs, this comprehensive effect fails to prove that the pathways regulated by ADM2 are all beneficial to osteogenic differentiation. Since PPARγ is a vital factor for adipogenic differentiation of BMSCs [55], ADM2 may potentially possess the ability to inhibit osteogenesis, contributing to its PPARγ-activating effect, which may lead to ADM2 inhibition of bone regeneration in non-diabetic individuals, thus affecting the indications for the application of ADM2 in clinical practice. Consequently, the effects and mechanisms of ADM2 on the osteogenic differentiation of BMSCs under normal conditions remain to be further investigated.

## Conclusions

This study demonstrates that ADM2 reverses AGE-induced M1/M2 imbalance partly through the PPARγ/IκBα/NF-κB signaling pathway and restores AGE-impaired osteogenic potential of BMSCs simultaneously, revealing ADM2 as a novel factor to accelerate bone regeneration under diabetic conditions during DO. Moreover, our study also provides a novel therapeutic strategy for diabetic patients undergoing DO, which suggests managing both inflammation and osteogenesis in parallel.

## Supplementary Information

**Additional file 1: Figure S1.** F4/80^+^ cells were identified as macrophages for further detection of CD86 and CD206 expression using flow cytometry analysis.

**Additional file 2.**

**Additional file 3.**

## Data Availability

The datasets used and/or analyzed during the current study are available from the corresponding author on reasonable request.

## Abbreviations

ADM2: Adrenomedullin 2
AGE: Advanced glycation end product
AKT: Activate protein kinase B
ALP: Alkaline phosphatase
Arg-1: Arginase1
ARS: Alizarin red S
BMD: Bone mineral density
BMDM: Bone marrow-derived macrophage
BMP-2: Bone morphogenetic protein 2
BMSC: Bone marrow mesenchymal stem cell
BSA: Bovine serum albumin
BV/TV: Bone volume/tissue volume
CGRP: Calcitonin gene-related peptide
CLR: Calcitonin receptor-like receptor
CT: Computed tomography
DAPI: 4′,6-Diamidino-2-phenylindole
DO: Distraction osteogenesis
EDTA: Ethylene diamine tetraacetic acid
ELISA: Enzyme-linked immunosorbent assay
E-modulus: Modulus of elasticity
FBS: Fetal bovine serum
GSK-3β: Glycogen synthase kinase-3 β
H&E: Hematoxylin-eosin
IGF-1: Insulin-like growth factor 1
IL-6: Interleukin-6
iNOS: Inducible nitric oxide synthase
IκBα: Nuclear factor kappa-light-chain-enhancer of activated B cells inhibitor alpha
MRC1: Macrophage mannose receptor 1
NF-κB: Nuclear factor kappa-light-chain-enhancer of activated B cells
OCN: Osteocalcin
OIM: Osteogenic induction medium
OSX: Osterix
P/S: Penicillin-streptomycin
PBS: Phosphate-buffered saline
PFA: Paraformaldehyde
PGL: Plasma glucose level
PPARγ: Peroxisome proliferator-activated receptor γ
qRT-PCR: Quantitative real-time polymerase chain reaction
RAGE: Receptor of advanced glycation end product
RAMP: Receptor-modifying protein
SD: Sprague Dawley
SO-FG: Safranin O-Fast Green
STZ: Streptozotocin
T1DM: Type 1 diabetes mellitus
TGF-β: Transforming growth factor β
TNF-α: Tumor necrosis factor α
Wnt: Wingless/integrated
