# Supplementary figures and images for: Adrenomedullin 2 improves bone regeneration in type 1 diabetic rats by restoring imbalanced macrophage polarization and impaired osteogenesis
Source: Stem Cell Res Ther. 2021 May 13;12:288. doi: 10.1186/s13287-021-02368-9 (PMC8117361; doi:10.1186/s13287-021-02368-9)

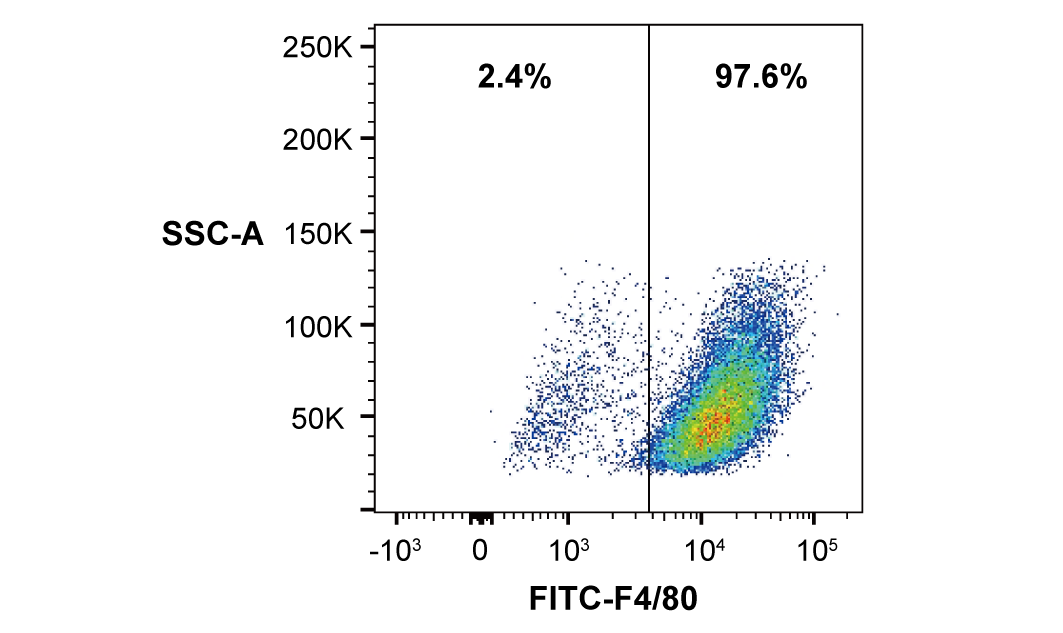

Supplement: Supplementary file 1 — Additional file 1: Figure S1. F4/80+ cells were identified as macrophages for further detection of CD86 and CD206 expression using flow cytometry analysis. [file 13287_2021_2368_MOESM1_ESM.png]
