# Supplementary material for: Adrenomedullin 2 improves bone regeneration in type 1 diabetic rats by restoring imbalanced macrophage polarization and impaired osteogenesis
Source: Stem Cell Res Ther. 2021 May 13;12:288. doi: 10.1186/s13287-021-02368-9 (PMC8117361; doi:10.1186/s13287-021-02368-9)
Supplement: Supplementary file 2 — Additional file 2. [file 13287_2021_2368_MOESM2_ESM.html]

xml version="1.0" encoding="utf-8"?CEFirst Log 

|  |
| --- |
| CEFirst Log |
| Version : 8.4.3 |
| USER | BGPC000028812P |

---

# Hyphenated Words

Please check the following hyphenated words for consistency:

- A–D [others 254, N-dash 1]
- AGE-RAGE [slash 5, hyphen 2]
- end-products [spaced 9, hyphen 1]
- Equal-volume [spaced 2, hyphen 1]
- gene-related [hyphen 4, spaced 1]
- ischemia-reperfusion [hyphen 2, slash 1]
- nuclear-translocation [spaced 5, hyphen 1]
- PPAR-γ [closed-up 34, hyphen 1]
- receptor-γ [spaced 3, hyphen 1]
- tetra-acetic [hyphen 1, closed-up 1]
- up-regulating [closed-up 1, hyphen 1]
- adipose-derived [hyphen 2]
- AGE-exposed [hyphen 1]
- AGE-impaired [hyphen 7]
- AGE-induced [hyphen 21]
- AGE-mediated [hyphen 2]
- anterior-posterior [hyphen 1]
- anti-BMP [hyphen 1]
- anti-CD [hyphen 1]
- anti-GAPDH [hyphen 1]
- anti-inflammation [hyphen 1]
- anti-inflammatory [hyphen 5]
- anti-IκBα [hyphen 1]
- anti-OCN [hyphen 2]
- anti-OSX [hyphen 1]
- anti-phosphorylated [hyphen 1]
- anti-PPARγ [hyphen 1]
- APC-conjugated [hyphen 1]
- atorvastatin-pretreated [hyphen 1]
- bone-regenerating [hyphen 1]
- chain-enhancer [hyphen 4]
- co-administration [hyphen 1]
- co-corresponding [hyphen 1]
- diabetes-induced [hyphen 1]
- DM-induced [hyphen 2]
- DM-related [hyphen 1]
- ECM-like [hyphen 1]
- El-Alfy [hyphen 1]
- El-Moghazy [hyphen 1]
- El-Mowafi [hyphen 1]
- E-modulus [hyphen 3]
- endothelial-cadherin [hyphen 1]
- enzyme-linked [hyphen 3]
- fibrous-like [hyphen 3]
- FITC-conjugated [hyphen 1]
- fluorophore-conjugated [hyphen 1]
- four-point [hyphen 1]
- glucose-induced [hyphen 1]
- glucose-lowering [hyphen 1]
- hematoxylin-eosin [hyphen 2]
- Ho-Shui [hyphen 1]
- HRP-streptavidin [hyphen 1]
- hyperhomocysteinemia-induced [hyphen 1]
- hypoxia-induced [hyphen 1]
- II-induced [hyphen 1]
- Image-Pro [hyphen 1]
- in-depth [hyphen 1]
- insulin-like [hyphen 2]
- kappa-light [hyphen 4]
- kinase-Akt [hyphen 1]
- L-ascorbic [hyphen 1]
- layer-wise [hyphen 1]
- L-c [hyphen 1]
- Lecka-Czernik [hyphen 1]
- Liraglutide-loaded [hyphen 1]
- Macrophage-mediated [hyphen 1]
- marrow-derived [hyphen 3]
- Melatonin-stimulated [hyphen 1]
- membrane-specific [hyphen 1]
- micro-computed [hyphen 1]
- Micro-CT [hyphen 2]
- mitogen-activated [hyphen 1]
- MSC-derived [hyphen 1]
- neo-callus [hyphen 1]
- neo-formed [hyphen 1]
- neo-osteogenesis [hyphen 1]
- NF-κB [hyphen 23]
- non-diabetic [hyphen 2]
- non-enzymatic [hyphen 1]
- O-Fast [hyphen 3]
- one-way [hyphen 6]
- osteogenesis-inducing [hyphen 1]
- osteogenesis-protective [hyphen 1]
- osteogenic-specific [hyphen 1]
- PE-conjugated [hyphen 1]
- penicillin-streptomycin [hyphen 2]
- phosphate-buffered [hyphen 1]
- PPARγ-activating [hyphen 1]
- products-induced [hyphen 2]
- pro-inflammatory [hyphen 3]
- proliferator-activated [hyphen 5]
- pro-regenerative [hyphen 1]
- P-value [hyphen 1]
- Q-l [hyphen 1]
- qRT-PCR [hyphen 6]
- RAGE-aptamer [hyphen 1]
- real-time [hyphen 4]
- receptor-gamma [hyphen 1]
- receptor-like [hyphen 2]
- receptor-modifying [hyphen 2]
- re-establishing [hyphen 1]
- SDS-PAGE [hyphen 1]
- semi-quantified [hyphen 1]
- Sigma-Aldrich [hyphen 3]
- signal-regulated [hyphen 1]
- SO-FG [hyphen 3]
- TGF-β [hyphen 8]
- Three-dimensional [hyphen 1]
- TNF-α [hyphen 8]
- Toll-like [hyphen 1]
- two-tailed [hyphen 1]
- week-old [hyphen 1]
- X-ray [hyphen 2]
- X-rays [hyphen 1]
- α-MEM [hyphen 1]
- β-catenin [hyphen 2]
- β-glycerophosphate [hyphen 1]
- μm-thick [hyphen 2]

---

Copyright © 2013-2021 SPi Global, Chennai, India
